# Riders on the storm: loggerhead sea turtles detect and respond to a major hurricane in the Northwest Atlantic Ocean

**DOI:** 10.1186/s40462-020-00218-6

**Published:** 2020-07-27

**Authors:** Leah M. Crowe, Joshua M. Hatch, Samir H. Patel, Ronald J. Smolowitz, Heather L. Haas

**Affiliations:** 1 Integrated Statistics under contract to the Northeast Fisheries Science Center, National Marine Fisheries Service, National Oceanic and Atmospheric Administration, 166 Water Street, Woods Hole, MA 02543 USA; 2 Northeast Fisheries Science Center, National Marine Fisheries Service, National Oceanic and Atmospheric Administration, 166 Water Street, Woods Hole, MA 02543 USA; 3 Coonamessett Farm Foundation, 277 Hatchville Road, East Falmouth, MA 02536 USA

**Keywords:** Satellite telemetry, Temperature, Behavioral response, Dive behavior, Cold pool, Mid-Atlantic bight, Extreme weather event

## Abstract

**Background:**

Extreme weather events, including hurricanes, have considerable biological, ecological, and anthropogenic impacts. Hurricane Irene caused substantial economic damage when it hit the Mid-Atlantic Bight (MAB) off of the eastern United States in August of 2011. The MAB is highly stratified during the summer when a strong thermocline separates warm, surface water from deep, cold water, and this oceanographic phenomenon makes modeling hurricane strength difficult. Loggerhead sea turtles (*Caretta caretta*) forage in the MAB primarily during the stratified season and their dive behavior to the bottom allows them to experience the oceanographic conditions of the entire water column.

**Methods:**

In this study, we analyzed the movements and dive behavior of juvenile and adult-sized loggerhead sea turtles (*n* = 18) that were foraging in the MAB as Hurricane Irene moved through the region. The satellite tags deployed on these turtles transmitted location data and dive behavior as well as sea surface temperature (SST) and temperature-depth profiles during this time.

**Results:**

Behavioral and environmental shifts were observed during and after the hurricane compared to conditions before the storm. During the hurricane, most of the turtles (*n* = 15) moved north of their pre-storm foraging grounds. Following the storm, some turtles left their established foraging sites (*n =* 8) moving south by 7.3–135.0 km, and for the others that remained (*n* = 10), 12% of the observed dives were longer (0.54–1.11 h) than dives observed before the storm. The in situ data collected by the turtle-borne tags captured the cooling of the SST (Mean difference = 4.47°C) and the deepening of the thermocline relative to the pre-storm conditions.

**Conclusions:**

Some of the loggerhead behavior observed relative to a passing hurricane differed from the regular pattern of seasonal movement expected for turtles that forage in the MAB. These data documented the shifts in sea turtle behavior and distribution during an ecosystem-level perturbation and the recorded in situ data demonstrated that loggerheads observe environmental changes to the entire water column, including during extreme weather events.

## Background

Extreme weather events, including hurricanes, have considerable biological, ecological, and anthropogenic impacts [1, 2], and the risk of loss increases as more people and resources interact with these disasters [2]. Hurricanes occur seasonally within each ocean basin, and have become more intense overtime, with this trend expected to continue [3]. Hurricane Irene hit the Mid-Atlantic Bight (MAB) of the United States’ eastern seaboard in August 2011, causing 49 human deaths and cost an estimated $15.8 billion (USD) in damages, mostly because of widespread flooding [4].

During the Atlantic hurricane season, the oceanography in the MAB is dynamic [5] and difficult to model [6] because a Cold Pool forms beneath a warm, surface layer [7–9]. Hurricane Irene was preempted by a 6°– 11°C decrease in sea surface temperature (SST; i.e. ahead-of-eye cooling) and a deepening of the thermocline by more than 15 m [10], primarily due to hurricane forced vertical mixing which was not accurately accounted for in hurricane modeling [10, 11]. The forecast for Hurricane Irene anticipated higher wind speeds that would have kept it along its projected path, but instead, the cooled surface waters decreased the strength of the storm before it made landfall, causing the storm to stall in areas that were unprepared [11]. Although the forecast models for Irene did not account for ahead-of-eye cooling, this phenomenon has occurred for all tropical cyclones that have interacted with the stratified conditions in the MAB since 1985 [10].

In addition to the impacts on the oceanography and human population, tropical storms and hurricanes can cause changes in the behavior of marine species. American lobsters (*Homarus americanus*) moved out of estuarine waters and into coastal waters during the passing of Hurricane Bob [12]. Sea snakes (*Laticauda* spp.) and juvenile blacktip sharks (*Carcharhinus limbatus*) moved into deeper waters as hurricanes and tropical storms caused barometric pressure to drop [13, 14]. Several shark species were temporarily displaced from study areas during the landfall of tropical storms [15], and increased emigration and daily movement rates of gray triggerfish (*Balistes capriscus*) were highly correlated with wave orbital velocity during the passing of two hurricanes [16]. Spotted seatrout (*Cynoscion nebulosus*) spawning was potentially delayed because of decreased water temperature due to a hurricane [17]. Finally, Eastern brown pelicans (*Pelecanus occidentalis carolinensis*) were observed sheltering in coastal regions during hurricanes to possibly allow for protection and rest [18].

There are few examples of sea turtle interactions with large storm events. Both a tagged loggerhead (*Caretta caretta*) and hawksbill (*Eretmochelys imbricata*) nesting female became more active when overlapping with extreme storms [19, 20]. Early tagging efforts of loggerhead sea turtles revealed some interaction between storms and two nesting females, but the data transmissions were thought to have been negatively impacted by the weather [21]. Adult loggerheads may have left their breeding areas in response to decreasing barometric pressure, a possible signal of worsening conditions [22]. Juvenile hawksbill turtles moved to deeper waters during two consecutive intense hurricane events in the Caribbean [23]. Modeling studies show that hurricanes can disrupt the migratory routes of younger sea turtles, potentially pushing them into unsuitable habitats [24, 25].

Foraging juvenile and adult loggerheads occur along the shelf in the MAB between May and September [26], overlapping substantially with the Atlantic hurricane season (June – November) [27] and the period when the Cold Pool is present (approximately May – October) [7]. The oceanography of the MAB is likely to play an important role in hurricane behavior and disaster preparation in the future, and this has encouraged the use of gliders to collect in situ data as storms work their way up the Northwest Atlantic Ocean [9]. Loggerhead sea turtle morphology and foraging behavior make them good observers of oceanographic variables throughout the entire water column where they forage [8, 28], and the data derived from turtle-borne data loggers are currently an under-utilized resource that has the potential to improve forecasting models [29].

In this study, we analyzed loggerhead sea turtle behavior in the MAB and the environmental conditions observed from turtle-borne satellite tags in relation to Hurricane Irene. We determined that as the storm passed, the tagged turtles altered their dive behavior and movement patterns. These observed changes in behavior can provide insight into turtle reactions to other perturbations. The turtle-borne tags also recorded the environmental changes to the water column as the hurricane passed through the MAB, highlighting these data as a valid resource for weather forecasting models alongside other in situ data sources.

## Methods

Loggerhead sea turtles were captured in early June and late July 2011 in the MAB with a large dip net. Netted turtles were transferred to a chartered commercial fishing vessel for sampling and tag attachment. Turtles were measured and weighed in compliance with the United States Endangered Species Act permitting requirements.

Satellite relay data loggers from the Sea Mammal Research Unit at the University of St. Andrews were attached to the carapace with a two-part epoxy. Tag parameterization was designed to allow for a lifespan achieving approximately 13 months [8]. For full details on handling and capture protocols as well as the parameterization of the tags used in this study, see Patel et al. [8]. A random sample of temperature-depth and dive data were transmitted [30], and recorded dives began when the tag was at or below 1.5 m depth for at least 20 s until it was dry or above 1.5 m depth. Tags were programmed to include both Fastloc-GPS and Argos locations for approximately the first three months of deployment, and Argos throughout the lifespan of the tag.

Location data from Argos and Fastloc-GPS data were filtered and combined into a single dataset. Only location quality classes 1–3 were retained for Argos positions, and Fastloc-GPS data were filtered for locations that were obtained using five or more satellites [31] where the residuals were greater than 0 and less than 25. These location data were combined and a speed filter with a maximum rate of 10 km h^− 1^ was applied to all location data [32]. Locations were mapped to ensure the lower resolution Argos locations fell within the path of the higher resolution Fastloc-GPS positions, and any visually erroneous points were excluded [33]. Data were compiled and analyzed using R 3.5.1 [34] and the *dplyr* [35], geosphere [36], *lubridate* [37], and *plyr* [38] R packages.

Hurricane Irene first formed on 21 August 2011 and was absorbed on 30 August 2011 [4]. Using the best track from the National Hurricane Center [39], the storm’s position at each location of a tagged turtle was determined using linear interpolation according to the following:

$$ \tilde{x},\tilde{y}=\left(1-\alpha \right)\left({x}_1,{y}_1\right)+\alpha \left({x}_2,{y}_2\right) $$

where (*x̃*, *ỹ*) is the interpolated storm location, (*x*_1_, *y*_1_) is the beginning location of the storm, (*x*_2_, *y*_2_) is the ending location of the storm, and α is the proportion of regular interval time between the timestamp of (*x*_1_, *y*_1_) and the timestamp associated with the tagged turtle’s location. The shortest distance along an ellipsoid between the tagged turtle and the interpolated track of Hurricane Irene was then calculated.

**Fig. 1:**
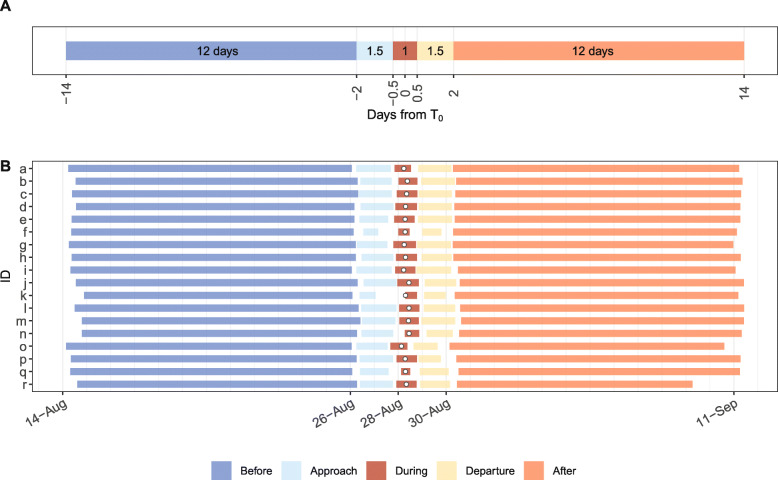
**a** The five hurricane phases were divided relative to the time when the turtle and the hurricane were closest to each other (*T*_0_) as follows: *Before* (14–2 days before *T*_0_), *Approach* (2–0.5 days before *T*_0_), *During* (0.5 days before–0.5 days after *T*_0_), *Departure* (0.5–2 days after *T*_0_), and *After* (2–14 days after *T*_0_). The *Approach* and *Departure* phases provided 1.5 day buffers around the *During* phase to determine clear *Before* and *After* phases. The length (days) of each phase is displayed within each colored section of the bar. **b** The range of the dates within each phase for each turtle are characterized by the colored sections of the horizontal bars. Each turtle had a different *T*_0_, and each turtle-borne tag transmitted at different times, therefore, the hurricane phases covered slightly different time periods per turtle. *T*_0_, which occurred at some time on 28 August 2011 for all turtles, is indicated by the white circle within the red, *During* section

Data derived from turtle-borne tags that were within 100 km of Hurricane Irene’s path at their closest point (*T*_0_) were included in this analysis. This distance ensured the inclusion of shelf-foraging turtles that were within the scope of the hurricane (approximately 180 km wide) as it passed through the MAB [4, 39]. Location data, temperature-depth profiles, and dive data two weeks before and after *T*_*0*_ were compiled to obtain pre- and post-storm conditions and behaviors. These data were divided into five phases: *Before*, *Approach*, *During*, *Departure*, and *After* where the *Approach* and *Departure* phases provided 1.5 day buffers around the one day *During* period to distinguish clear 12 day *Before* and *After* phases (Fig. 1a). Because each turtle had a unique *T*_*0*_ and each tag transmitted at different times, the hurricane phases covered slightly different time periods for each turtle (Fig. 1b).

### Behavioral data

The pre-storm foraging range for each turtle was calculated as the latitudinal range between the 5th and 95th percentile of the latitudinal extent for the turtle’s movements three to six weeks prior to *T*_*0*_. There was less focus on longitudinal movements as loggerhead sea turtles within the region typically remain resident during the foraging season and take seasonal long-distance latitudinal migrations when arriving and leaving the MAB [26]. Box plots of turtle latitude were calculated for the *Before, During,* and *After* phases to assess the extent of latitudinal movement. Turtle movements were characterized by analyzing the interquartile range (the middle 50% of the distribution) of latitudinal movements within the hurricane phases relative to the foraging range—if these overlapped, a turtle was considered to have remained in its foraging range.

Dive behaviors were assessed by analyzing daytime (0600–2000 EDT) dive duration and maximum dive depth for individuals that remained in their foraging ranges in order to investigate dive behavior relative to local changes in the habitat. Daytime diving was specifically chosen because changes to dive behavior could substantially impact the availability of turtles at the surface during line-transect surveys, and subsequent abundance estimates [40]. Dive duration relative to maximum dive depth was analyzed to better understand the composition of the type of dive behavior exhibited in each phase. To compare dive behavior relative to the hurricane, dive duration and maximum dive depth data were pooled and summarized for these turtles in the *Before* and *After* phases.

### Environmental data

Turtles that remained in their foraging ranges along a similar latitudinal range were considered the best observers of the hurricane, and, therefore, SST data and temperature-depth profiles collected from the turtle-borne tags of these individuals were analyzed. Data collected nearshore (< 5 km) were excluded to ensure the comparison of temperatures was only across shelf waters and not from shallow, warmer regions. SST values were collected at 2.0 m depth and data collected from all turtle-borne tags were compiled together to gain a perspective on the conditions the turtles were experiencing throughout the passage of Hurricane Irene. SST data were pooled and summarized for these turtles in the *Before* and *After* phases to further compare these changes. Full water column profiles were analyzed relative to each phase for each turtle to understand how the local water column may have been affected by the hurricane. Temperature-depth profiles reaching within 15% of ETOPO1 Ice Surface Global Relief Model [41] bathymetry were considered full water column profiles as described in Patel et al. [8]. Bathymetry was analyzed using the *raster* [42] and *rgdal* [43] R packages.

**Fig. 2:**
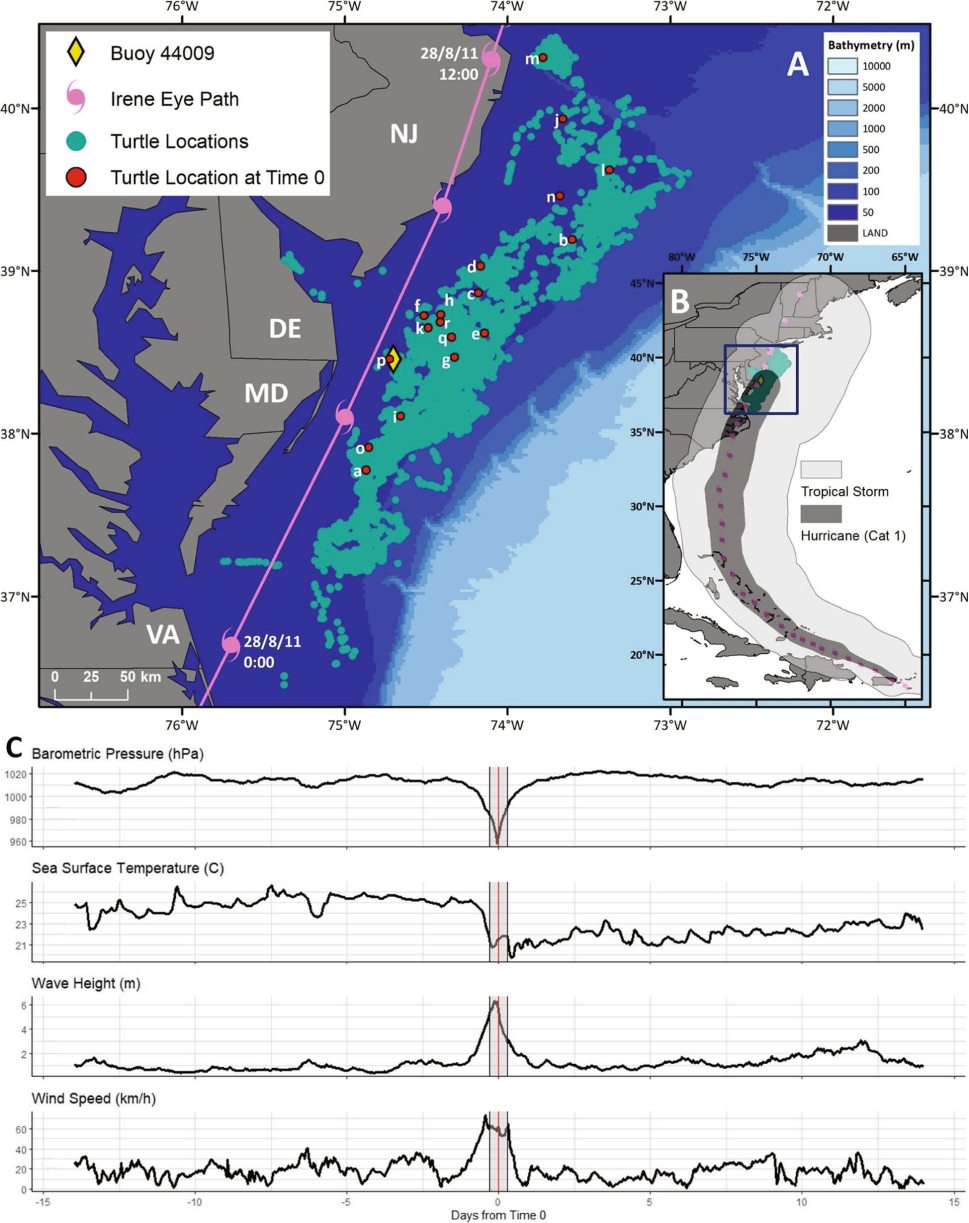
All locations transmitted from tagged turtles between 14 August and 11 September 2011 (green circles), the track of Hurricane Irene (pink symbols and lines, UTC) [39], and the environmental conditions two weeks before and after the hurricane recorded by buoy 44009 (yellow diamond) [45]. **a** The position where the hurricane and each turtle were closest to each other (*T*_0_) occurred on 28 August 2011 for all turtles and are indicated by the red circles. Buoy 44009 was close to the hurricane path and approximately in the middle of the turtle positions within this time period. **b** The inset map, denoted by the blue square, illustrates the wind speeds associated with Hurricane Irene. The dark grey indicates Category 1 hurricane strength (119–153 km h^-1^), and the light grey indicates tropical storm strength (63–119 km h^-1^). **c** Barometric pressure, sea surface temperature, wave height, and wind speed data gathered by buoy 44009. The red vertical line denotes *T*_0_, the grey shaded area bordered by the grey vertical lines denotes the 0.5 day period on either side of *T*_0_ spanning the time frame of the *During* phase

Turtle-borne tag locations and the hurricane’s path were plotted using ArcGIS 10 [44] (Fig. 2a, b). The National Oceanic and Atmospheric Administration’s National Data Buoy Center Station 44009 is positioned at 38.457°N, 074.702°W [45], halfway north to south between the turtles’ extent. Due to its location and availability of data, this buoy was identified as the best source of traditional in situ weather data (barometric pressure, SST, wave height, and wind speed) for this region during Hurricane Irene (Fig. 2c).

**Fig. 3:**
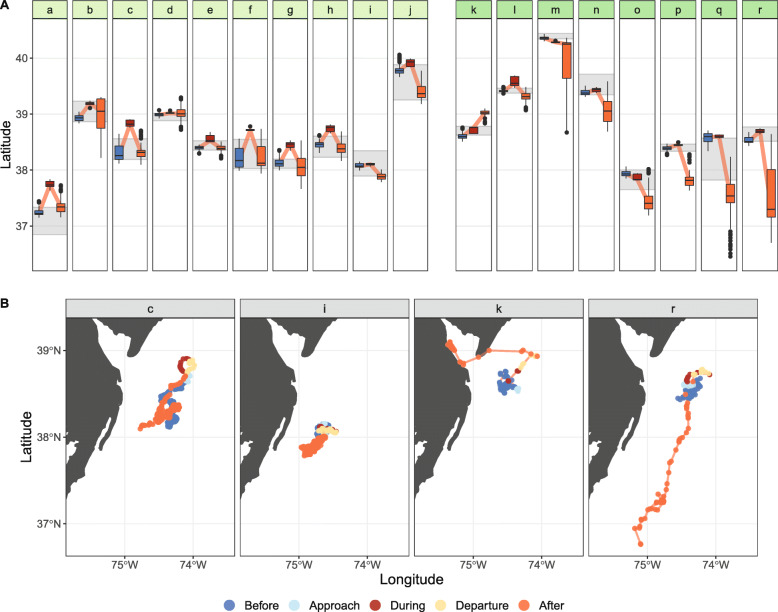
**a** The latitudinal movements of tagged turtles in the *Before*, *During*, and *After* phases relative to the passing of Hurricane Irene. For each of these phases, the colored box represents the interquartile range (25th to 75th percentile), the horizontal black line within the colored box represents the median value, the upper and lower whiskers represent values that fall within 1.5x the interquartile range, and the black dots represent the outlying points [46]. The grey shaded area for each turtle represents its foraging range which spanned between the 5th and 95th percentile of the latitudinal extent for the time period three to six weeks prior to *T*_0_. Turtles *a – j* (light green) remained in their foraging range as the interquartile range within the *After* phase overlapped with their foraging range, while turtles *k – r* (darker green) left their foraging grounds after the hurricane passed. The light red line connects the medians between each phase to highlight the overall movement pattern. **b** Example turtle movements in all phases: *c*) This animal was one of the turtles that revisited their foraging grounds after moving north in the *During* and *Departure* phases; *i*) This animal did not leave its pre-storm foraging area in the *During* phase, but did move a bit south in the *After* phase; *k*) This animal did not return to its foraging ground, and instead was the only animal in this study that moved inshore; *r*) One of the animals that did not return to its foraging ground, and instead went south by almost 2° of latitude in the *After* phase

**Fig. 4:**
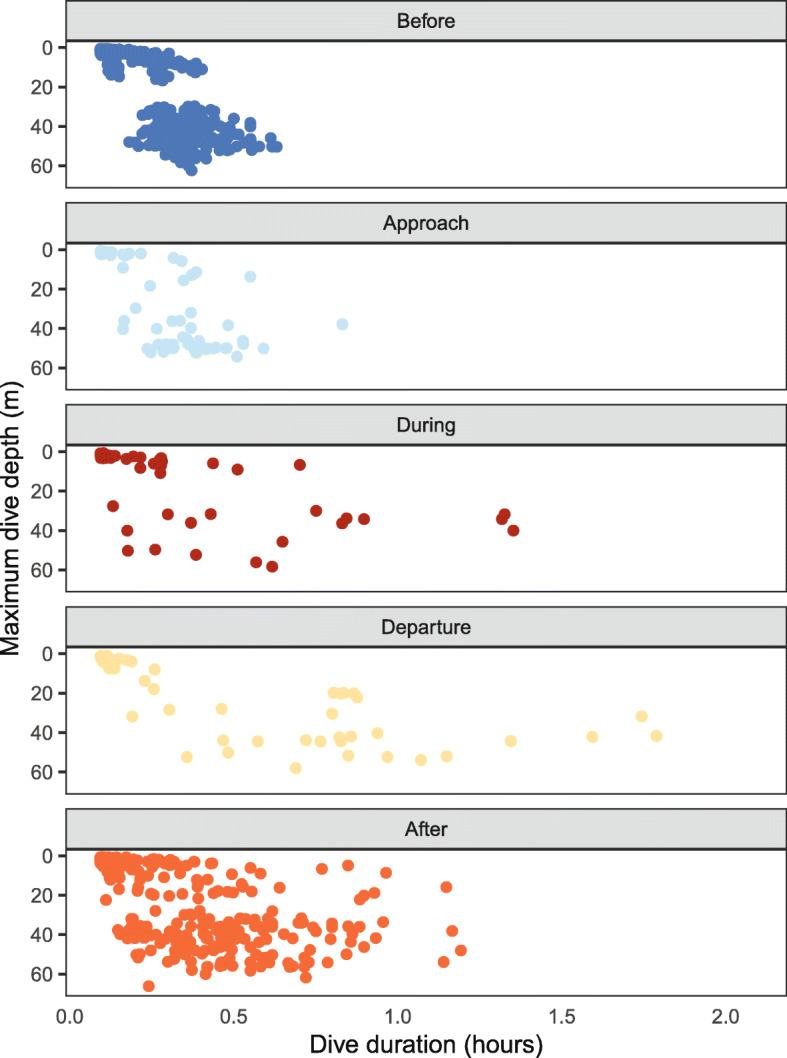
Dive duration (hours) relative to the maximum dive depth (m) throughout all phases for turtles that remained in their foraging grounds after Hurricane Irene passed *(a – j)*

**Fig. 5:**
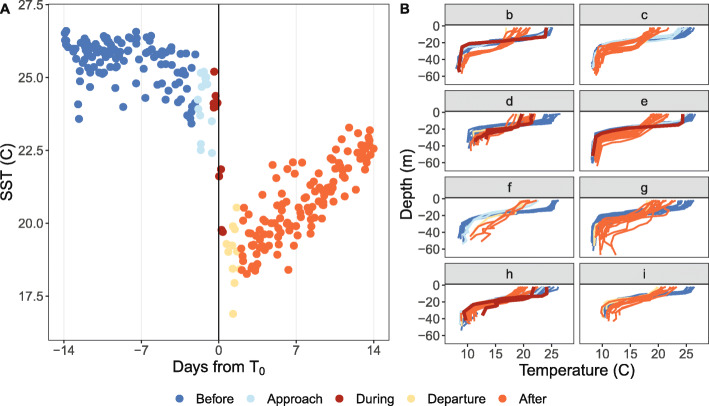
In situ water temperature data collected from turtle-borne tags *b – i*. These turtles remained in their foraging areas in the *After* phase and were along a similar latitudinal range (between 37.68°N and 39.30°N). **a** Sea surface temperature values collected at 2 m depth and **b** full water column (within 15% of the bottom) temperature-depth profiles throughout all phases

Figures 1, 2c, 3, 4 and 5 were created using the *ggplot2* [46] and *ggpubr* [47] R packages.

## Results

Eighteen turtles were within 9.4–80.1 km of the eye of Irene at their closest points (Fig. 2a). Their straight carapace length (SCL), measured from nuchal notch to posterior marginal tip, ranged from 58.1 to 87.0 cm (*n* = 17, Mean = 73.0 cm; Table 1). Six turtles (*e, g, h, m, p,* and *r*) measured a SCL greater than 75 cm, which was consistent with adult size according to Avens et al. [48]. Additionally, the curved carapace lengths measured from nuchal notch to posterior marginal tip (*n* = 18, Mean = 78.9 cm) were used to identify the life stage corresponding to potential reproductive output according to Murray [49]. Six classifications are within this scheme ranging from Stage I to Stage V (adult)—Stage IV is divided into two categories, IVa and IVb, where Stage IVb has a wide range of reproductive potential [49]. Turtles in this study represented middle and later juvenile Stages III (*n* = 7), IVa (*n* = 7), and IVb (*n* = 4) (Table 1).

**Table 1:**
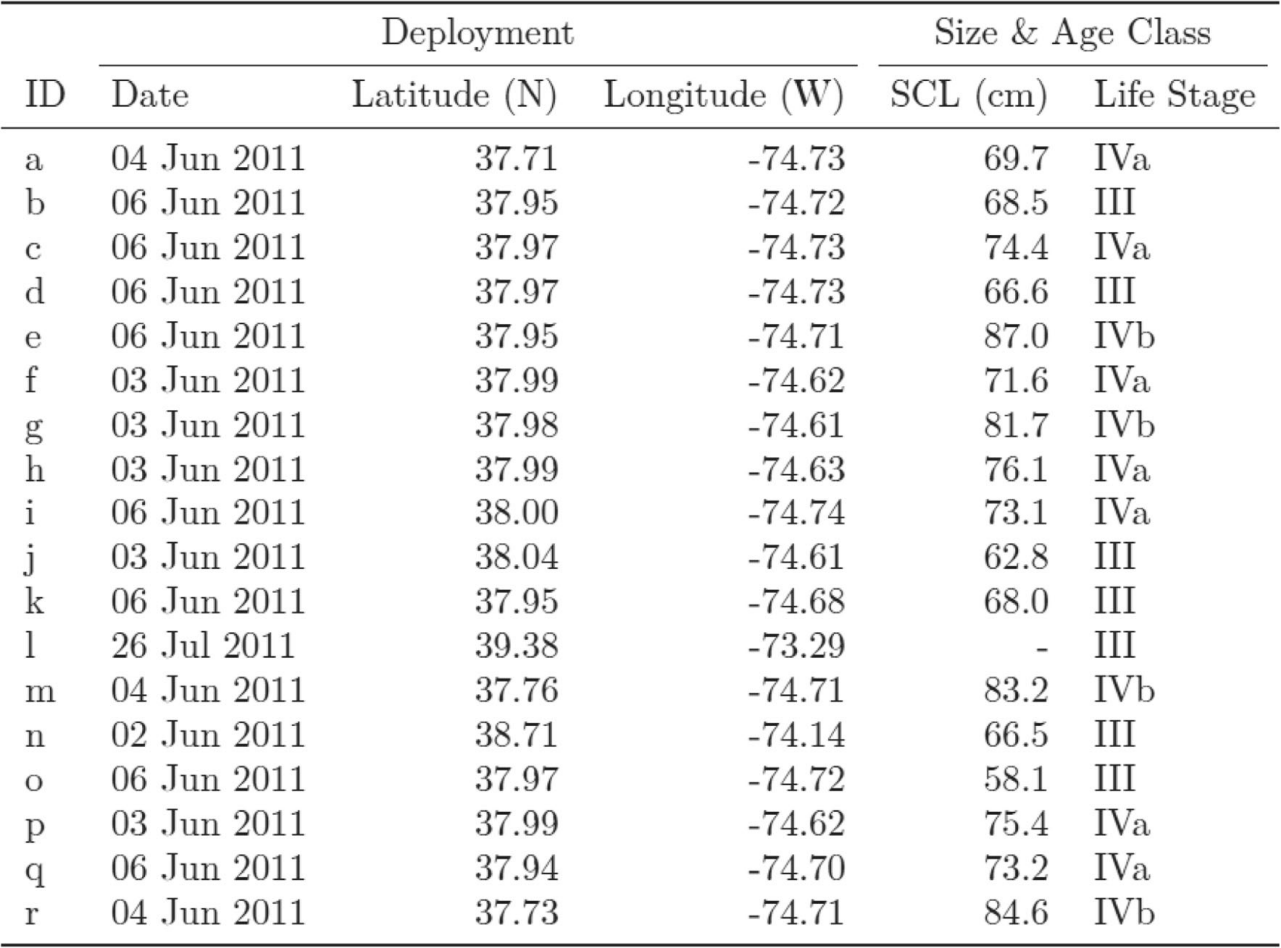
Deployment details, straight carapace length (SCL), measured from the nuchal notch to posterior marginal tip, and size class for each tagged turtle used in this analysis. Life stages III and IV are mid and later stage juveniles, respectively [49]

The turtles were closest to Hurricane Irene’s path (*T*_*0*_) on 28 August 2011 for all individuals when the severity of the storm ranged between a hurricane and a tropical storm. Within the five phases defined here (*Before, Approach, During, Departure,* and *After*), 5573 positions were transmitted from all turtle-borne tags between 14 August 2011 and 11 September 2011. Fastloc-GPS transmissions were received throughout the study period, and similar numbers of positions came from each source in the *Before* and *After* phases (ARGOS: *Before* = 720, *After* = 698; Fastloc-GPS: *Before* = 1782, *After* = 1617; Supplementary Fig. 1A, Additional file 1). Of the 108 turtle-phase combinations (Supplementary Fig. 1B, Additional file 1), there were only six instances where more locations came from Argos than Fastloc-GPS (*k* and *r* in the *Before*, *a* and *k* in the *Approach*, and *d* and *k *in the *During* phases), and there were six cases where there was only Fastloc-GPS data. An additional interactive map can be used to further investigate the tracks, including the location source, for the turtles throughout this study period [see Additional file 2].

### Behavioral data

Turtle pre-storm foraging areas occurred between 36.80°N and 40.47°N and were calculated from 6708 positions. This period occurred between 17 July 2011 and 14 August 2011 for all turtles except turtle *l* where the included data began the day after it was tagged, 27 July 2011. The latitudinal extent of each area varied amongst individuals and ranged between 9.7 and 82.7 km (Fig. 3a).

Turtle residency *Before* the hurricane was primarily consistent with each turtle’s pre-storm foraging area, but movement out of the foraging area was observed within the *During* and *After* phases of the hurricane. In the *During* phase, all but three of the turtles (*m, o,* and *q*) moved north, and about two thirds of the turtles (*a, c, d, e, f, g, h, j, l,* and *q*) had interquartile ranges north of their foraging areas—one turtle (*m*) had an interquartile range south of its foraging area. Turtles moved 0.3 (turtle *q*)—62.1 km (turtle *c*) between the median latitudes of the *Before* and *During* phases.

Despite some temporary northward movement *During* the hurricane, the interquartile range in the *After* phase was at least partially within the pre-storm foraging area for 10 turtles (*a – j*; Fig. 3a, light green), and completely outside of it for eight turtles (*k – r*; Fig. 3a, darker green), which had mostly moved south. A comparison of median latitudes of the 10 turtles that remained in their foraging areas showed that all of them went north in the *During* phase and then came south in the *After* phase (Fig. 3a, *e.g.* Fig. 3b, turtle *c*). Two of the turtles that remained did not leave their foraging area in the *During* phase (e.g. Fig. 3b, turtle *i*). Of the eight turtles that went out of their foraging grounds after the hurricane, one of them went inshore (Fig. 3b, turtle *k*), and the rest went south, traveling 7.3–135.0 km between the southern limit of their pre-storm foraging site and the median latitude of the *After* phase (e.g. Fig. 3b, turtle *r*).

Dive duration and maximum dive depth changed as the hurricane passed the animals remaining in their foraging areas (*n* = 1185 total dives, Fig. 4). Longer dive durations were observed in the *During* phase and continued into the *Departure* phase when the longest dive was observed (1.70 h). While dive duration shortened moving from the *Departure* to the *After* phase, they were still longer than dives in the *Before* and *Approach* phases. *Before* the passage of the hurricane, all but one dive was less than 0.54 h. *After* the hurricane, approximately 12% of the dives, taken by nine of the 10 turtles, exceeded 0.54 h, including a few that exceeded one hour (Max = 1.11 h, Fig. 4). The *Before* and *After* phases had similar numbers of dives (*Before*: *n* = 381; *After*: *n* = 366), and the dive duration was longer (hours; *Before*: Mean = 0.19, SD = 0.13; *After*: Mean = 0.23, SD = 0.24) and maximum dive depth was shallower (m; *Before*: Mean = 26.33, SD = 20.57; *After*: Mean = 20.60, SD = 19.91) in the *After* phase. However, shallow and deep dives were observed throughout all phases of the hurricane (Fig. 4).

### Environmental data

Turtles *b – i* were considered good hurricane observers as these animals remained in their foraging grounds and were also within a similar latitudinal range (between 37.68°N and 39.30°N). Data collected in this region (*n* = 354) showed SST dropping at *T*_0_ followed by a warming throughout the *After* phase (Fig. 5a). The number of SST data points collected in the *Before* and *After* phases were similar (*Before*: *n* = 249; *After*: *n* = 251), and the SST was cooler in the *After* phase (°C; *Before*: Mean = 25.14, SD = 1.20; *After*: Mean = 20.67, SD = 1.36). Additionally, temperature-depth profiles (*n* = 225) were compiled for these turtles relative to all five phases (Fig. 5b). These profiles revealed changes in the thermocline relative to the passing of the hurricane, and for all turtles, deeper and cooler thermoclines were observed in the *After* phase.

*T*_0_ for the weather buoy was also identified as 28 1August 2011. A decrease in barometric pressure and SST and an increase in wave height and wind speed after *T*_0_ was recorded during this period (Fig. 2c) consistent with the passing of a Category 1 hurricane [4, 10].

The location, dive, and temperature-depth data generated and analyzed in this study are available in Additional file 3.

## Discussion

Hurricane Irene tracked along a large portion of the United States eastern seaboard that is valued for recreation, shipping, fishing, seismic exploration, and wind farm development. It is also an important habitat for many protected species [50], and supports densely populated coastal communities [51]. Due to this confluence of potentially conflicting interactions between humans and protected species, this region is highly managed in order to reduce harmful anthropogenic impacts. As the region experiences a rising intensity in ecosystem perturbations, including hurricanes [3], effective management becomes harder as animals may become displaced in space and time.

Ecosystem-level perturbations can cause turtles to alter their routine behavior by direct effects or through habitat modifications. Here we show that sea turtle distribution and dive behavior change with an extreme weather event. Elsewhere, storm degraded habitats have possibly resulted in shifts in loggerhead foraging areas [52]. During a marine heatwave, loggerheads moved into shallower regions where the destruction of seagrass beds may have revealed short-term access to new benthic forage [53]. Anthropogenic perturbations can also illicit direct or indirect changes to sea turtle behavior. Multiple low-sample-size studies suggest the possibility that sound may trigger a response in turtles [54, 55]. Dredge operations were associated with changes in flatback sea turtle (*Natator depressus*) distribution and dive behavior, possibly due to noise impacts and changes to the benthos [56]. Seismic activity, windfarm development, and marine recreation may impact sea turtle distribution and dive behavior directly or through habitat alterations [54, 57–59]. The long-term, cumulative impacts of climate and anthropogenic impacts on sea turtle behavior and distribution in the MAB warrant consideration [57, 60] and may have legacy effects [53] for sea turtle research and management.

### Behavior

Loggerhead sea turtles overlap spatiotemporally with extreme weather events, and turtle dive behavior did not return to pre-storm behavior within at least two weeks following a storm. The latitudinal displacement and changes in loggerhead dive behavior are the consequences of either an acute perturbation, an alteration of larger scale oceanographic conditions, or both. The behavioral changes observed in this study are an opportunistic look at neritic turtle behavior relative to a hurricane, and it is currently unclear what aspect of the environment (i.e. sound from increased wind, shifts in barometric pressure, altered currents, lower SSTs, deepened thermocline, or disrupted prey fields) led to the observed behavior.

The turtles that left their foraging ranges moved south earlier than would be expected from normal patterns of seasonal movement, and earlier than the seasonal water column cooling [7, 8, 26]. Hurricane Irene passed through the MAB at the end of August 2011 when there was still more than a month of the foraging season remaining [26] (Supplementary Fig. 2 Additional file 1), and more than a month before the Cold Pool normally dissipates due to the autumn turnover [7, 8]. Despite this, nearly half of the turtles in this study vacated their pre-storm foraging areas and moved south by as far as 135 km after the hurricane—a distance that is nearly 25% of a typical southward migration to the coastal waters of North Carolina [26]. While in the MAB, loggerheads usually exhibit seasonal home range fidelity and occupy a SST thermal range between 18.2°–29.2°C [28]. Although the water temperatures quickly cooled due to the storm, the turtles that remained in their pre-storm foraging ranges did not experience SSTs outside of typically occupied temperatures (Fig. 5).

Turtles that stayed in their foraging ranges took longer dives after the hurricane. The difference in mean dive duration before and after the hurricane may not be biologically significant, but the increased range suggests that the storm, and its impacts on the water column, may have had an effect. The changes in dive duration observed with the passing of the hurricane are similar to behavior changes observed with seasonal decreases in SST [61]. Loggerhead behavior is likely sensitive to these changes in thermal conditions, as research has identified that a 2°C difference in SST can alter dive behavior [62, 63]. These changes in dive behavior could have been triggered by the transformation of the entire water column, including the decrease in SST [64], disruption of the thermocline, and the weakening of the overall stratification [65].

The northward movements for most of the turtles during the hurricane aligned with the direction of the dominant along-shelf surface current associated with the storm’s passing [10]. In the 12 h after the eye of Irene passed the MAB, the current velocity at up to 20 m depth was 0.4–0.8 m s^-1^ moving northward [10]. The horizontal movement of loggerhead sea turtles in all life stages are influenced by surface currents, either by physical mechanisms [66, 67] or by learned experiences [68]. Smaller turtles that swim slower than currents are able to influence their overall trajectory and survival success by purposeful alignment and short periods of active swimming [67, 69–71]. Juvenile loggerheads are capable of escaping current patterns [69, 72], but powerful currents can alter the course of even a strong, adult turtle [73]. The movement north of most of the turtles in this study during the hurricane could possibly be a tactic to conserve energy by purposeful alignment [69, 72, 74].

A shift in sea turtle distribution and diving behavior can influence the calculations used in creating abundance estimates from aerial surveys, and abundance estimates that assume constant sea turtle behavior through time may appear more accurate than they are [38, 75]. As other studies have reported, neglecting the variability of environmentally influenced dive behavior can have substantial effects on density estimates [75]. Analysis of visual survey data collected after extreme weather events or other perturbations should consider that turtles may be triggered to leave an area and/or alter their dive patterns.

### Animal-borne & weather buoy in-situ environmental observations

Tagged turtles that remained in their foraging regions during Hurricane Irene effectively recorded the SST drop as well as the vertical mixing of the stratified MAB. The water temperature data from the turtle tags were consistent with weather buoy and glider observations during this period, but more spatially extensive than other oceanographic data sources [10]. Stratified environments are challenging to model, and more data from in situ sources are needed and being sought to improve hurricane forecasting in these types of regions [9, 11]. The ahead-of-eye cooling is a characteristic of Atlantic hurricanes that come through the MAB during the stratified summer period [10], and this aspect has proven to be a major limiting factor to accurately model storms as well as prepare regions for hurricane damage. We show in this study that turtle-borne tags collect additional in situ data on the MAB during a time of year that is the perfect storm of turtle foraging season, stratification and formation of the Cold Pool, as well as the Atlantic hurricane season.

## Conclusions

Consistent with Patel et al. [11], turtle-borne data can provide in situ oceanography data in the seasonally stratified MAB as loggerhead sea turtles observe the entire water column profile, including during extreme weather events. This study gave one example of how loggerhead behavior and distribution changed during an ecosystem perturbation, suggesting that we cannot assume consistency through time. Future analysis of turtle behavior should consider the dynamic environments in which they live.

## Supplementary information

**Additional file 1.** Location source for tagged turtles and previously observed proportions of tagged turtles in the Mid-Atlantic Bight by month.

**Additional file 2.** Interactive map of turtle tracks before, during, and after Hurricane Irene.

**Additional file 3.** Location source for tagged turtles and previously observed proportions of tagged turtles in the Mid-Atlantic Bight by month.

## Data Availability

Data generated and analyzed during this study are included in this published article [Additional file 3].

## Abbreviations

cm: Centimeter
EDT: Eastern Daylight Time
Fig.: Figure
GPS: Global positioning system
h: Hour
km: Kilometers
m: Meters
MAB: Mid-Atlantic Bight
°N: Degrees North
*n*: Sample size
SCL: Straight carapace length
SD: Standard deviation
SST: Sea Surface Temperature
*T*_0_: Time when the turtle is at its closest point to Hurricane Irene
USD: United States Dollars
°W: Degrees West
degrees C: Degrees
Celsius: Seconds
