# Supplementary material for: Riders on the storm: loggerhead sea turtles detect and respond to a major hurricane in the Northwest Atlantic Ocean
Source: Mov Ecol. 2020 Jul 27;8:32. doi: 10.1186/s40462-020-00218-6 (PMC7385951; doi:10.1186/s40462-020-00218-6)
Supplement: Supplementary file 1 — Additional file 1. Location source for tagged turtles and previously observed proportions of tagged turtles in the Mid-Atlantic Bight by month. [file 40462_2020_218_MOESM1_ESM.pdf]

Supplementary, Additional file 1

Riders on the storm: loggerhead sea turtles detect and respond to a major hurricane in the Northwest Atlantic Ocean

Leah M. Crowe*[a], Joshua M. Hatch[b], Samir H. Patel[c], Ronald J. Smolowitz[c], Heather L. Haas[b]

*Corresponding Author

[a]Integrated Statistics under contract to the Northeast Fisheries Science Center, National Marine Fisheries Service, National Oceanic and Atmospheric Administration, 166 Water Street Woods Hole, MA 02543 USA. [b]Northeast Fisheries Science Center, National Marine Fisheries Service, National Oceanic and Atmospheric Administration, 166 Water Street Woods Hole, MA 02543 USA. [c]Coonamessett Farm Foundation, 277 Hatchville Road East Falmouth, MA 02536 USA.

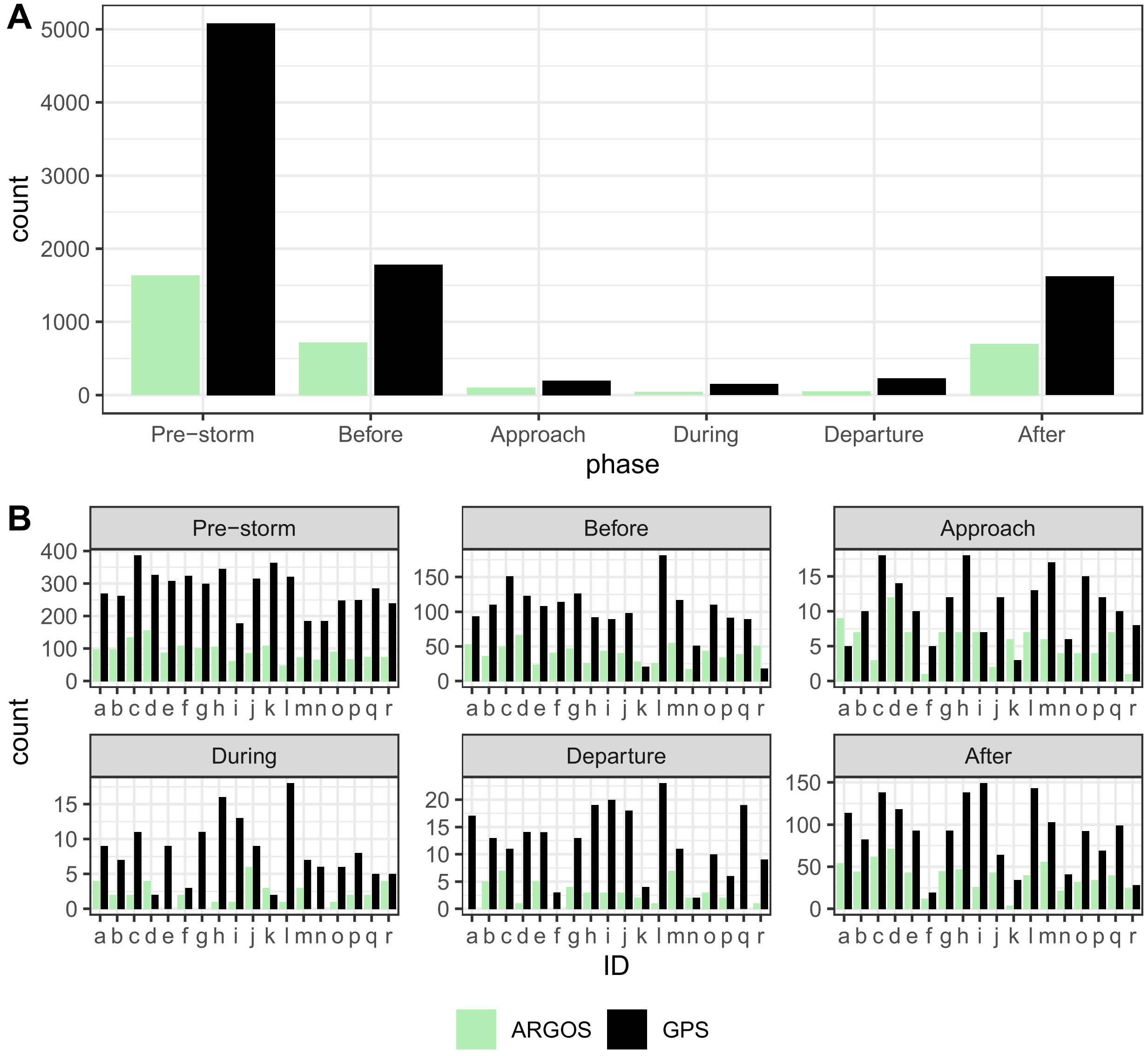

Supplementary Fig. 1. The number of positions transmitted by ARGOS and Fastloc-GPS (GPS) sources in each phase (A) as well as for each turtle-borne tag (B).

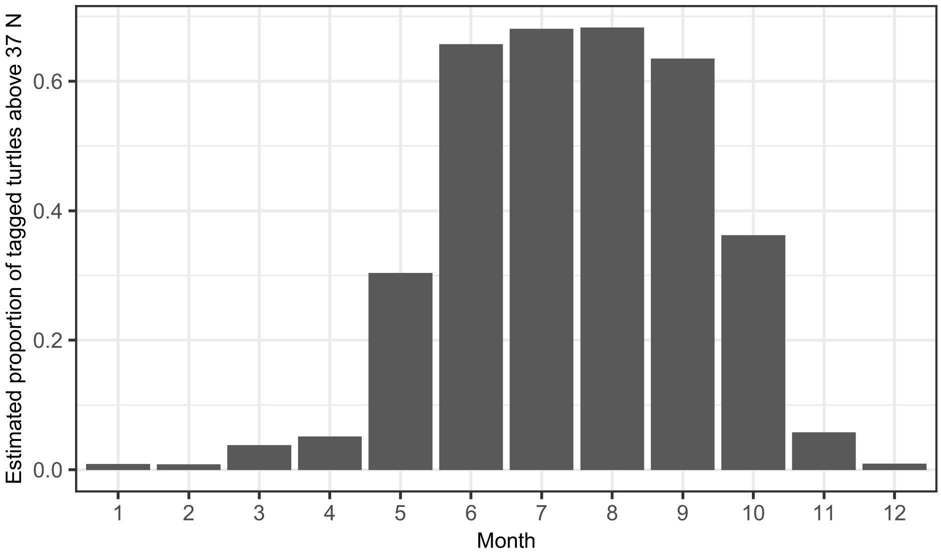

Supplementary Fig. 2. The proportion of tagged turtles that were in the MAB in each month from data collected between 2004 and 2016. Turtles are foraging in the MAB in all months between June and September in similar proportions. The proportion for each month was summed for all grid cells at or above 37°N in the shapefiles available from the modeling results from Winton *et al.* [26] available at https://www.fisheries.noaa.gov/new-england-mid-atlantic/endangered-species-conservation/sea-turtle-ecology-and-population-dynamics.
