# Supplementary material for: Riders on the storm: loggerhead sea turtles detect and respond to a major hurricane in the Northwest Atlantic Ocean
Source: Mov Ecol. 2020 Jul 27;8:32. doi: 10.1186/s40462-020-00218-6 (PMC7385951; doi:10.1186/s40462-020-00218-6)
Supplement: Supplementary file 3 — Additional file 3. Location source for tagged turtles and previously observed proportions of tagged turtles in the Mid-Atlantic Bight by month. [file 40462_2020_218_MOESM3_ESM.zip › Croweetal2020__Ridersonthestorm_datadocumentation.pdf]

Documentation of available data that accompanies 'Riders on the storm: loggerhead sea turtles detect and respond to a major hurricane in the Northwest Atlantic Ocean'

Leah M. Crowe*[a], Joshua M. Hatch[b], Samir H. Patel[c], Ronald J. Smolowitz[c], Heather L. Haas[b]

July 14, 2020

*Corresponding Author

[a]Integrated Statistics under contract to the Northeast Fisheries Science Center, National Marine Fisheries Service, National Oceanic and Atmospheric Administration, 166 Water Street Woods Hole, MA 02543 USA. [b]Northeast Fisheries Science Center, National Marine Fisheries Service, National Oceanic and Atmospheric Administration, 166 Water Street Woods Hole, MA 02543 USA. [c]Coonamessett Farm Foundation, 277 Hatchville Road East Falmouth, MA 02536 USA.

The data included here have been filtered according to the methods, and the following definitions apply to all datasets:

- **ID** = identification letter assigned in this study
- **TIME2DAY0** = the time before (negative) or after (positive) when the turtle and the hurricane were at their closest point, T_0
- **phase** = the phase assignment based on the time from T_0 (TIME2DAY0)

Location data: Croweetal2020_Ridersonthestorm_location.csv

This dataset includes location data from ARGOS and Fastloc-GPS sources for the *Before* through *After* phases.

- **D_DATE** = UTC. For ARGOS data: The time assigned to the location by ARGOS. For Fastloc-GPS data: the time that the fix was obtained
- **LQ** = Location quality where values 1–3 indicate ARGOS positions and 9 indicates Fastloc-GPS
- **LAT** = Position estimate (WGS 84 datum)
- **LON** = Position estimate (WGS 84 datum)

Dive data: Croweetal2020_Ridersonthestorm_dive.csv

This dataset includes daytime dive data (duration and maximum dive depth) from turtles that remained in their foraging grounds after Hurricane Irene passed (turtles *a* – *j*).

- **DS_EDT** = the dive start (Eastern Daylight Time) when the threshold depth was crossed
- **divehr** = the dive duration (hours)
- **MAX_DEP** = The maximum dive depth recorded (decibars)

Temperature-depth data: Croweetal2020_Ridersonthestorm_tempdep.csv

This dataset includes temperature-depth data from turtles that remained in their foraging grounds after Hurricane Irene passed and were within a similar latitudinal range (turtles *b* – *i*)

- **END_DATE** = The time of the end of the upcast
- **TEMP_DBAR** = A comma-separated list of the pressure coordinates of the (pressure,temperature) profile (decibars)
- **TEMP_VALS** = A comma-separated list of the temperature coordinates of the (pressure,temperature) profile (°C)
